# Application of Electrochemical Biosensors for Determination of Food Spoilage

**DOI:** 10.3390/bios13040456

**Published:** 2023-04-03

**Authors:** Krisztina Majer-Baranyi, András Székács, Nóra Adányi

**Affiliations:** 1 Food Science Research Group, Institute of Food Science and Technology, Hungarian University of Agriculture and Life Sciences, Villányi út 29-43, H-1118 Budapest, Hungary; 2 Agro-Environmental Research Centre, Institute of Environmental Sciences, Hungarian University of Agriculture and Life Sciences, Herman Ottó út 15, H-1022 Budapest, Hungary

**Keywords:** electrochemical biosensors, food spoilage, pathogens, contaminants, biomarkers, enzyme-based techniques, immunosensors, monitoring

## Abstract

Food security is significantly affected by the mass production of agricultural produce and goods, the growing number of imported foods, and new eating and consumption habits. These changed circumstances bring food safety issues arising from food spoilage to the fore, making food safety control essential. Simple and fast screening methods have been developed to detect pathogens and biomarkers indicating the freshness of food for safety. In addition to the traditional, sequential, chemical analytical and microbiological methods, fast, highly sensitive, automated methods suitable for serial tests have appeared. At the same time, biosensor research is also developing dynamically worldwide, both in terms of the analytes to be determined and the technical toolkit. Consequently, the rapid development of biosensors, including electrochemical-based biosensors, has led to significant advantages in the quantitative detection and screening of food contaminants. These techniques show great specificity for the biomarkers tested and provide adequate analytical accuracy even in complex food matrices. In our review article, we summarize, in separate chapters, the electrochemical biosensors developed for the most important food groups and the food safety issues they can ensure, with particular respect to meat and fish products, milk and dairy products, as well as alcoholic and non-alcoholic beverages.

## 1. Introduction

The expansion of international and interregional trade in human food increases the risk of transportation of pollutants over long distances and associated food spoilage. Food security is significantly affected by the mass production of agricultural produce and goods, the growing number of imported foods, and changes in food consumption habits [1]. With lifestyle changes, new eating habits have emerged, such as the consumption of raw foods and an increasing demand for ready meals and fast foods to save costs and time [2]. These factors highlight food safety issues due to food spoilage, making food safety control measures even more essential. Therefore, simple and fast screening methods must be developed to detect pathogens in such products including biomarkers indicating food freshness to ensure food safety [3]. Achievements in the early detection of food pathogens and spoilage microorganisms throughout the supply chain can help prevent food spoilage, mass food losses, and health risks [2].

Fast and accurate analytical tools are essential for ensuring food safety and avoiding food spoilage caused by contamination and pathogens. While significant developments have been achieved in analytical techniques, traditional or modern methods with advanced instrumentation are often limited by long analysis times, expensive and laborious sample preparation, and the need for highly trained personnel. These factors render traditional methods overly laborious for use in routine quality assurance systems. In addition to modern food testing methods such as high-performance liquid chromatography (HPLC), gas chromatography (GC), mass spectrometry (MS), HPLC-MS, GC-MS, atomic and molecular spectroscopy, and nuclear magnetic resonance (NMR) spectroscopy, various biological and molecular biological methods have also emerged. Examples of these include immunoanalytical methods, such as enzyme-linked immunoanalytical methods (ELISA, EIA), and molecular biological methods, such as polymerase chain reaction (PCR)-based methods including real-time PCR (RT-PCR) and randomly amplified polymorphic DNA PCR (RAPD-PCR) methods.

In the past three decades, fast, highly sensitive automated methods suitable for serial tests were also introduced in addition to traditional, sequential, chemical analytical and microbiological methods. In parallel, biosensor research that began in the 1960s is also dynamically developing worldwide both in terms of the analytes to be determined and the technical toolset. Accordingly, the rapid development of biosensors, including electrochemical biosensing, has led to significant advantages in the quantitative detection and screening of food contaminants. These biosensors show high specificity towards the biomarker investigated and also provide adequate analytical accuracy even in complex food matrices [4]. Various types of biosensors have gained practical applications, including enzyme-based sensors, immunosensors, and affinity sensors. Rapid tests are also available although only for on-site inspections for food safety. The sensitivity and sufficiently low limit of detection (LOD) of these methods enable the rapid detection of microbiological contamination in food, helping to prevent epidemic-like diseases.

**Figure 1:**
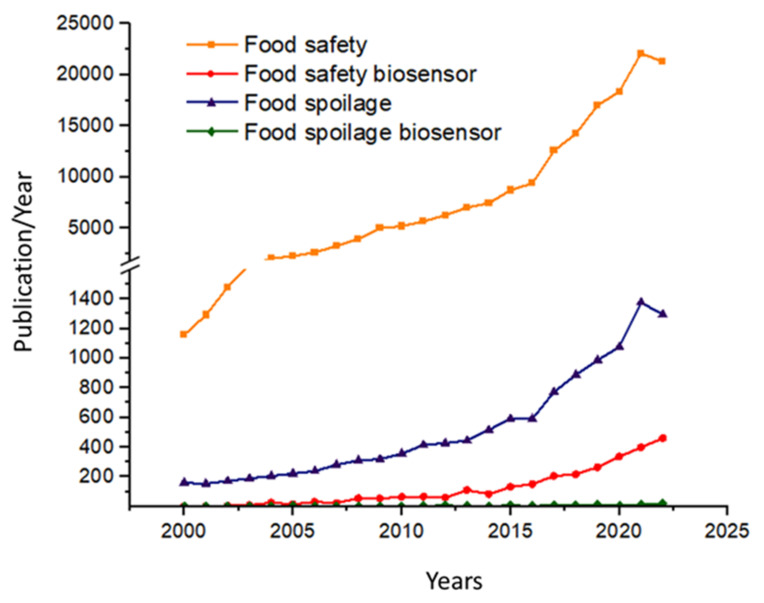
Number of papers published annually during the past two decades found using the keywords “Food safety”, “Food safety biosensor”, “Food spoilage”, and “Food spoilage biosensor” from the Web of Science database (accessed on 21 March 2023).

Food safety is a pivotal issue nowadays, primarily relating to the aspects of toxins, mycotoxins, and chemical residues, yet food spoilage receives significantly less attention. In this publication, we report on biosensor developments published in recent years related to food spoilage, primarily highlighting the diversity of practical implementations. The continuous development of biosensors, as well as applications related to food safety and food spoilage, has brought enormous attention to the new field of biosensor research within analytical chemistry, which is also reflected in the published literature (Figure 1). By searching in the ScienceDirect database for keywords such as “Food safety” and “Food safety biosensor”, it could be established that the number of publications on the topics since 2000 has been about 380,000 and about 12,000, respectively, while searching for “Food spoilage” and “Food spoilage biosensor” yielded only about 30,000 and 1600, respectively.

## 2. Biosensors

**Figure 2:**
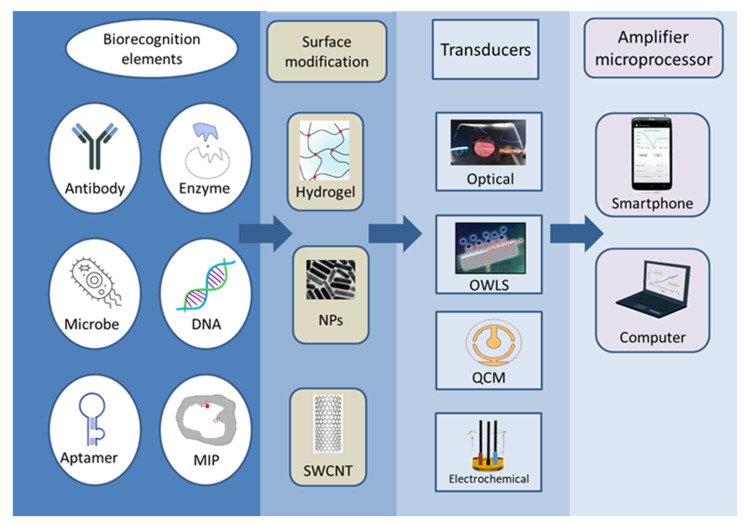
The diversity of the construction possibilities of biosensor types.

According to the definition proposed by IUPAC, a biosensor is an independent integrated device that provides quantitative or semi-quantitative analytical information by means of a biological recognition system in direct spatial contact with the transducer [5]. Based on this structure, biosensors must be distinguished from other analytical measurement tools, and due to its multiple uses, they must also be discerned from single-use tests and devices. During biosensor measurement, the sample first comes into contact with the surface of the sensor receptor; the sensor registers the physical or physicochemical changes that occur during the interaction between the substance to be measured and the receptor; and the detected signal is converted, electronically stored, and evaluated [6]. The operation and efficiency of the sensor are determined by two main components: the biological recognition part and the signal conversion unit. The two are jointly responsible for the selectivity and sensitivity of the sensor. Numerous combinations of biosensors can be devised (Figure 2), many of which have been investigated in the last nearly fifty years. High sensitivity, low LOD, specificity, reproducibility, and robustness are just some of the properties expected from biosensors.

The first biosensor was designed by Clark and Lyons in 1962 for determining the concentration of glucose. They immobilized glucose oxidase and detected the loss of oxygen in the enzymatic reaction using an oxygen electrode overlaid with a semipermeable dialysis membrane [7,8,9]. The enzymatic oxidation of glucose takes place on the sensor surface resulting in localised oxygen loss, leading to a drop in the current at the oxygen electrode. This specific and sensitive amperometric setup was initially devised for medical purposes but has since led to the development of a wide range of devices that use the novel technique of biosensors and apply different surface modification, biorecognition, amplification, and signal processing methods.

A variety of biorecognition elements can be incorporated into biosensors, including enzymes, aptamers, antibodies, nucleic acids, cells, tissues, and molecularly imprinted polymers (MIPs) [10]. The selection of the biologically active substance depends on the analyte to be determined as well as the related detector. Enzymes are protein-type biocatalysts, which are stable and specific, and can be easily immobilised on the surface of the receptor. Antibodies are proteins produced by the immune system that show a high affinity and specificity for their target molecules. Aptamers are artificial oligonucleotides that are designed to bind with a high affinity to various target molecules only acting on specific locations of the molecule. MIPs are template-shaped cavities in polymer matrices with appropriate selectivity and high affinity. MIPs can be used instead of natural biomolecules because they are extremely stable, cheap, and easy to prepare [11].

**Figure 3:**
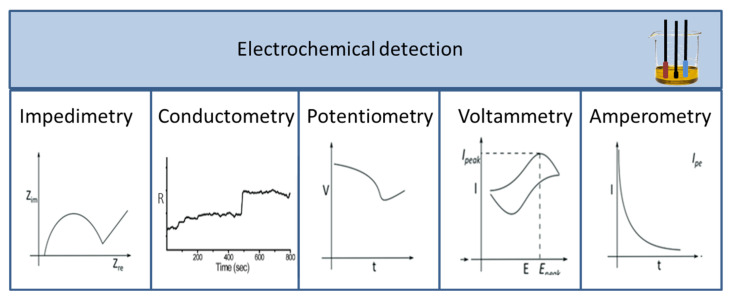
Different types of electrochemical sensors and their characteristic curves.

Signals produced during a biological, biochemical, or chemical reaction are measured with different detectors. As a result, a wide range of detectors is used in biosensors, which are described and compared in numerous scientific articles. Among the detectors, the most common ones are optical [12,13,14,15,16] and electrochemical sensors, including potentiometric, voltammetric, and conductivity measurements [17,18,19,20]. According to their operation, sensors can be divided into labelled and label-free techniques. Labelling substances include enzymes, nanospheres (such as Au or TiO_2_), and fluorescent or chemiluminescent dyes followed by the appropriate detection method, while detection may operate based on mass changes for example in surface plasmon resonance (SPR) spectroscopy and optical waveguide lightmode spectroscopy (OWLS), or using piezoelectric quartz crystal microbalance (QCM) instruments. Electrochemical biosensors include traditional techniques (Figure 3), such as potentiometry, voltammetry, amperometry, impedance spectroscopy, and various field-effect transistor-based applications, as well as promising new approaches such as nanowire- or magnetic nanoparticle-based biosensing [21].

During potentiometric measurements, the potential difference between an indicator and a reference electrode is determined if no significant current occurs between them. The transducer can be an ion-selective electrode (ISE), which is an electrochemical sensor based on thin films or selective membranes as recognition elements. The most common potentiometric devices are pH electrodes and ion- or gas-selective (CO_2_, NH_3_) electrodes. Their response depends significantly on the buffer capacity and ionic strength of the sample [5]. A silver–silver chloride (Ag–AgCl) secondary electrode is most often used as a reference electrode. The generated potential, therefore, depends linearly on the logarithm of the activity of the ion to be measured in the solution and of the ion for which the electrode is selective within a given ion’s activity range.

Potential differences between the indicator and reference electrodes are proportional to the logarithm of the chemical activity (or concentration) of the ion, as described by the Nernst equation. The equation expresses the equilibrium electrode potential (*E*) in terms of the actual ion activities. Since the activities of molecules or ions dissolved in solution are assumed to be approximately the same as their molar concentrations, the equation for electrode potential is often written using concentrations:

(1) $$E=E^{0}\prime +\frac{RT}{nF}ln\left(\frac{C_{O}}{C_{R}}\right)$$

where *E*^0^′ is the formal potential for the electrochemical couple, *R* is the universal gas constant (8.314 J/mol K), T is the absolute temperature (K), n is the number of electrons, *F* is the Faraday constant (96,485 C/mol), and *C_O_* and *C_R_* are the concentrations of the dissolved molecules or ions in the oxidised and reduced forms, respectively, at the surface of the electrode [5].

Potentiometric biosensors have a very simple structure and are reliable, but at the same time, their response time and regeneration time are relatively long. A potentiometric sensor for the detection of histamine (His) was developed in which MIP nanoparticles were embedded in poly(vinyl chloride) (PVC) membranes [22]. A label-free potentiometric immunosensor was investigated with a gold nanoparticle (AuNP) polymer inclusion membrane (PIM) as a pipette tip-type glassy carbon electrode (GCE) for the detection of *Salmonella typhimurium* [23].

### 2.1. Voltammetry

Voltammetry is an analytical procedure in which a specific voltage profile is applied to a working electrode as a function of time, and the current produced by the system is measured. The measurement requires a well polarisable working electrode and a reference electrode. By measuring the analyte under theoretically ideal conditions/environments, the peak value of the measured current is in the linear potential range, meaning that it is directly proportional to the bulk concentration of the electroactive substance. As the potential can be varied in different ways, there are different methods for voltammetry, the most widely used forms being cyclic voltammetry (CV), stripping voltammetry (SV), square wave voltammetry (SWV), and pulse or differential pulse voltammetry (DPV) [21].

A sandwich electrochemical immunosensor was developed for the determination of *Salmonella* species, where antibodies were immobilised on a AuNP-modified screen-printed carbon electrode (AuNP-SPCE). The signal produced by a horseradish peroxidase (HRP)-labelled secondary antibody was detected by CV [24]. Histidine was detected by an MIP-based sensor, where the MIP beads were attached to the electrode surface by sol–gel immobilisation and adsorptive SV was used in differential modes [25]. A multiwalled carbon nanotube-polyallylamine-modified screen-printed electrode (MWCNT-PAH/SPE)-based immunosensor was developed for simultaneous determination of *E. coli*, *Campylobacter*, and *Salmonella* in milk samples. The mixture of antibodies was immobilised on the modified electrode surface. Secondary antibodies were conjugated with different nanocrystal tracers (CuS, PbS, and CdS) and square wave anodic stripping voltammetry (SWASV) was employed to measure the released metal ions [26]. A square wave voltammetry-based sensor was adapted for hypoxanthine (Hx), xanthine (Xa), and uric acid (Ua) measurement in fish samples using an edge plane pyrolytic graphite electrode (EPPGE) [27]. An MIP sensor was developed to determine His, where DPV was applied with glassy carbon electrodes (GCEs) having AuNPs on the surface [28].

### 2.2. Amperometry

Amperometry is based on the measurement of current resulting from the oxidation or reduction of an electroactive substance in a biochemical reaction [21]. It is usually performed by maintaining a constant potential on a Pt-, Au-, or C-based working electrode or an electrode array with respect to a reference electrode, which can also serve as an auxiliary electrode when the current is low. The generated current directly correlates with the concentration either of the electroactive material or the rate of production/use within the biocatalytic layer [21]. Amperometric measurements are mainly used in combination with enzyme- or immunosensor-based techniques. Chronoamperometry is also a known and popular detection method for different types of biosensors, where a square wave potential is applied to the working electrode and a steady state current is measured as a function of time [29]. The expansion or reduction of the diffusion layer at the electrode causes the alterations in the current measured.

Amperometric Xa biosensors have been developed on different types of electrodes. For example, a layer-by-layer electrode architecture on GCE based on hybrid nanomaterials [30], or xanthine oxidase (XOD), was covalently immobilised onto citrate-capped silver nanoparticles (AgNPs) deposited onto a disposable Au electrode through a self-assembled monolayer (SAM) [31]. An amperometric biosensor for Xa determination was developed based on the immobilisation of XOD onto a carbon paste electrode (CPE) modified with a hybrid nanocomposite film [32]. Dalkiran et al. used an SPCE electrode modified with tyrosinase (TYR), poly-L-lysine (PLL), and an Fe_3_O_4_ nanoparticle chitosan composite for tyramine (Tra) determination [33]. A chronoamperometric sensor was developed by carboxymethylated cashew gum (CMCG) film electrodeposited onto a Au surface for the immobilisation of polyclonal anti-*Salmonella* antibodies [34].

### 2.3. Conductometry

Conductometry is an electrochemical measurement method suitable for monitoring changes in the conductivity during various interactions, including enzymatic reactions and biological membrane processes. Conductometric devices using microelectrodes can follow numerous enzymatic reactions and the reactions of biological membrane receptors as a result of changes in ion concentrations [5]. Since the sensitivity of the measurement is hindered by the conductivity of the sample solution, differential measurements are usually performed by testing the sample solution between the sensor with the enzyme and the sensor without the same enzyme.

### 2.4. Electrochemical Impedance Spectroscopy

Electrochemical impedance spectroscopy (EIS) combines the analysis of resistive and capacitive properties of materials based on the perturbation of an equilibrium system by a sinusoidal excitation voltage signal. EIS can be used for the analysis of interfacial properties on the electrode surface relating to biorecognition events and explore mass transfer, charge transfer, and diffusion processes, to study intrinsic material properties or specific processes that influence conductance, resistance, or capacitance.

The spectroscopic measurement of electrochemical impedance involves measuring the AC impedance of the system, which represents a resistance used as a model for the resistance measured in direct current circuits, but in an alternating current circuit, as a function of frequency. The excitation signal is a small-amplitude sine wave, and the response is a current that differs in both amplitude and phase. Data are usually plotted on a Nyquist diagram, which shows the changes in the real and imaginary parts of the impedance as a function of frequency, and on Bode diagrams, which show the absolute value of the impedance as a function of frequency. The electrical properties of the measured system are usually modelled with substitute electronic circuits that contain passive elements, such as resistance and capacitance [35,36].

The advantage of EIS is that it allows for checking of the impedance of the biological reaction taking place on the surface of electrodes in a wide frequency range. Numerous biomolecules have been used as detection elements with varying success as bioreceptors of impedimetric sensors [37].

A selective and sensitive immunosensor was developed for the detection of *Salmonella typhimurium* species using a specific surface antigen attached to SPCEs modified with graphene–graphene oxide (G–GO). The change in impedance response signals indicated the concentration of the *S. typhimurium* serovariant in fruit juice [38].

## 3. Monitoring of Spoilage in Meat, Fish, and Their Products

Meat is the most valuable livestock product, and over the last 20 years, the consumption of meat and meat products has increased by 18% worldwide, with further increases anticipated despite ecological consequences. Meat, meat products, and fish are highly perishable, and globally, 20% of the produced meat is wasted. In the European Union (EU), 14 million tonnes of meat are wasted annually, resulting in significant economic losses and environmental impacts. Therefore, the quality and safety control of these products is of utmost importance throughout the food chain, from farm to fork [39,40].

Meat spoilage is a complicated mechanism, as meat proteins are prone to different chemical, enzymatic, microbial, and environmental degradation, resulting in reaction products that are undesirable for human consumption. During the deterioration of meat, the presence of different microorganisms, the breakdown of proteins, fats, and carbohydrates results in unpleasant odours, off-flavours and slime formation, making the product unacceptable for human consumption [41]. Several chemical compounds such as biogenic amines (BAs), putrescine (Put), cadaverine (Cad), His, Tra, spermidine (Spd), spermine (Spm), tryptamine, total volatile basic nitrogen (TVB-N), trimethylamine (TMA), ammonia gas, and hydrogen sulphide, are produced via the actions of bacteria and enzymes as a result of protein decarboxylation and deamination. As the increase in the amount of these components is related to the deterioration of the meat, these substances are considered as quality and freshness indicators of meat and meat products [37]. Measurement of adenosine triphosphate (ATP) breakdown products has also been used as a marker of meat quality. Several techniques have been applied to assess meat quality and freshness, including standard microbiological methods and conventional analytical techniques as mentioned in Section 2. Although these techniques are routinely used in the field of quality assurance in the food industry, they are time-consuming, require trained personnel and expensive equipment for sample analysis, and are not appropriate for continuous monitoring [42]. Considering that these products have a short shelf life, the development of analytical methods that allow for the rapid screening of pathogens and spoilage indicators is critical to ensure food safety.

### 3.1. Enzyme-Based Biosensors for Histamine, Putrescine, and Tyramine

Enzyme-based electrochemical biosensors are the most commonly used techniques among biosensors for BA determination. As recognition elements, amine oxidases catalysing the oxidative deamination of primary amines, diamines, and substituted amines are used for the quantification of BAs, via the measurement of oxygen consumption or the hydrogen peroxide (H_2_O_2_) production. Biosensors based on amine oxidases are suitable only for total BA determination as their substrate specificity is broad. For the specific determination of different amines such as His, Tra, and Put, selective enzymes such as methylamine dehydrogenase, monoamine oxidases, or putrescine oxidase have to be used. The drawback of these sensors is that a very high operational potential has to be applied, resulting in high background currents and a drastic reduction in electrode selectivity when H_2_O_2_ production is detected. To overcome this problem, coupled biosensors combining amine oxidases and peroxidases as well as different modified electrode platforms have been developed, which are summarised in Table 1 [43,44,45,46].

An amperometric biosensor using amine oxidase and an SPCE modified with MnO_2_ was designed for BA determination in chicken meat samples [47]. The product of the enzymatic reaction, H_2_O_2_, was detected by amperometry at +400 mV. The LODs were determined to be 0.3 µM for Cad and Put and 3.0 µM for Tra and His. The sensor could be used for up to 40 runs (about 1400 injections), quantifying all four substrates.

Torre et al. presented an amperometric enzyme sensor based on an SPCE and diamine oxidase (DAO) for His determination in fish samples [48]. As a detection technique, chronoamperometry was used at a potential of −0.3 V. With this enzyme sensor setup, the linear measurement range was 1–75 mg/L in the fish extract and it could be reused for up to seven measurements.

A simple, miniaturised and low-cost sensor was recently developed for His analysis in tuna and mackerel samples [49]. The quantification of His was achieved by chronoamperometry (+ 0.2 V, 120 s) using hexacyanoferrate (III) as a redox mediator. The sensor was able to determine His in a concentration range between 5 and 75 mg/mL, with an LOD of 0.97 mg/L.

An amperometric biosensor based on an SPCE modified with DAO, graphene, and platinum nanoparticles (PtNPs) was introduced by Apetrei et al. for His detection in freshwater fish samples [50]. H_2_O_2_ produced during the enzymatic reaction was measured at a potential of +0.4 V. The calibration curve was linear in the His concentration range from 0.1 to 300 μM. The sensor showed good selectivity for His (with cross-reactivities of less than 10% for Car, Tra, and Put).

A tetrathiafulvalene (TFF)-modified SPCE-based monoamine oxidase (MAO) enzyme sensor was prepared for Put determination in zucchini and anchovy samples [51]. An electrode modification for amperometric detection enabled a substantially improved operational potential (+250 mV) compared to electrodes without the TFF mediator (+600 mV). The biosensors showed a linear response range from 16 to 101 μM and an LOD of 17.2  ±  4.6 μM for Put.

An amperometric MAO-based biosensor for the simultaneous determination of Put and Cad using two working electrodes connected in an array mode was presented [52]. The biosensor was developed on the basis of the TFF/SPCE electrode, as previously presented, combined with AuNPs. The measurements were carried out at a potential of +250 mV. The developed biosensor was used for the simultaneous determination of Put and Cad in octopus samples with a linear response range from 9.9 to 74.1 μM and 19.6 to 107.1 mM for Put and Cad, respectively [52].

MAO/ HRP- and DAO/HRP-based biosensors using SPCEs were reported for total BA determination in fish samples [43]. The linear response range for His was 0.2 up to 1.6 μM and from 0.4 to 2.4 μM for the DAO/HRP- and MAO/HRP-based biosensors, respectively, and the corresponding LODs were 0.18 ± 0.01 μM and 0.40 ± 0.04 μM.

In the work of Pérez et al., a DAO- and HRP-based His sensor was developed on an SPCE electrode surface modified with a polysulfone/carbon nanotube/ferrocene membrane [53]. Electrochemical measurements were carried out at a potential of −50 mV to minimize interference. The sensor exhibited high sensitivity, high storage stability, and excellent reproducibility, obtaining a dynamic measuring range from 3 × 10^−7^ to 2 × 10^−5^ M, with an LOD of 1.7 × 10^−7^ M.

A combination of enzymatic layers for specific BA determination was developed, by immobilising MAO, tyramine oxidase, and DAO enzymes with defined activities each on a separate screen-printed thick-film electrode to form an enzyme sensor array for the simultaneous determination of His, Tra, and Put [54]. LODs of 10 mg/kg for His and Tra and 5 mg/kg for Put, with linear ranges up to 200 mg/kg for His and Tra and 100 mg/kg for Put, could be achieved. The established sensor allowed a rapid quantitative determination of the three BAs requiring only a simple sample pre-treatment. The samples were extracted with 5% trichloroacetic acid, centrifuged, and then the collected supernatants were used directly for the enzyme sensor array after pH adjustment.

Similarly, for multiplex detection, amperometric enzyme sensor arrays were presented for the detection of His, Put, and Cad [57]. In the biosensor arrays, three differently modified SPCE working electrodes (Co(II)-phthalocyanine and modified Prussian blue, as well as an Os-wired HRP-modified electrode) were used, where DAO was immobilised on the electrodes using magnetic nanobeads as a support. The three sensors had low working potentials (+0.4 V, −0.1 V, and −0.05 V, respectively) and in all cases, the LODs for His, Put, and Cad were in the submicromolar to micromolar range. The sensor presented a broad linear range of two orders of magnitude, good reproducibility, high repeatability, and no interferences from other related compounds. The biosensor has been applied for BA determination in spiked and naturally spoiled fish.

A modified SPCE-based amperometric biosensor was developed for Spm and Spd determination [55]. On the surface of the modified electrode, polyamine oxidase or spermine oxidase enzymes were immobilised. H_2_O_2_ produced due to the enzymatic reaction was measured at +700 mV for the graphite electrode or −100 mV for the Prussian blue-modified SPCE. The biosensor using polyamine oxidase showed a linear range of 0.003–0.3 mM for Spm and 0.01–0.4 mM for Spd, while with the spermine oxidase-based biosensor, a linear range of 0.004–0.5 mM for Spm was obtained.

Gumpu et al. used a GCE modified with DAO on cerium oxide (CeO₂) nanoparticles to fabricate an amperometric biosensor for Put determination in the tiger prawn [56]. During the experiment, the produced H_2_O_2_ was measured by amperometry at a potential of 0.729 V. On the basis of the amperometric response, different calibrated models such as linear, Hill, and Michaelis–Menten functions were constructed. For calculation of Put concentrations in the tiger prawn, all three models were used from which Hill analysis showed an acceptable recovery with low relative standard deviation. The proposed biosensor system could be a useful tool for the quality control of tiger prawns.

Draz et al. developed a potentiometric biosensor for rapid and direct Tra determination in turbid and coloured food samples such as blue cheese, aged cheese, Egyptian pickled cottage cheese, and pickled herring. The sensor potential was linear within the concentration range of 1 × 10^−2^–7.74 × 10^−5^ M, with an LOD of 5.8 × 10^−5^ M for Tra [58].

### 3.2. Enzyme-Based Biosensors for Xanthine

After slaughter, meat loses its freshness due to autolysis and the breakdown of ATP. ATP and inosine monophosphate (IMP) dominate in fresh meat, which is one of the major factors contributing to its pleasant flavour. However, right after slaughter, ATP degrades into adenosine diphosphate (ADP), adenosine monophosphate (AMP), inosine 5-phosphate (IP), inosine, Hx, Xa, and Ua. The process is catalysed by XOD, which oxidizes Hx to form Xa and Us. During ATP degradation, the major metabolites formed are Hx and Xa, which impart a bitter off-taste as they accumulate gradually during storage. Thus, they can be used as indicators of freshness [42,59].

Thandavan et al. reported the development of a mediator-free biosensor using iron oxide (Fe_3_O_4_) nanoparticles and XOD for Xa determination as an indicator for fish freshness [60]. For determination, amperometry was used at a constant potential of 0.5 V. Based on their study, it was found that the biosensor was able to determine the concentration of Xa in the range of 0.4 to 2.4 nM. The LOD was found to be 0.4 nM with a quick response time of 2 s due to the enhanced electron transfer pathway by the XOD/Fe_3_O_4_/Au-modified electrode.

A novel amperometric biosensor for Xa determination was developed based on the immobilisation of XOD onto a CPE modified with a hybrid nanocomposite film [32]. The biosensor exhibited a linear working range from 0.2 to 36.0 μM with an LOD of 0.1 μM. The fabricated biosensor was successfully applied for the control of fish and chicken meat freshness.

Dolmaci et al. developed an amperometric biosensor for Hx determination in fish by immobilising XOD with pyrrole and polyvinylsulphonate on the surface of a Pt electrode [61]. The determination of Xa was based on the oxidation of Ua liberated during the enzymatic reaction on the surface of the electrode at +0.3 V. The linear working range of the enzyme electrode was 1.0 × 10^−7^–1.0 × 10^−3^ M and the LOD was 1.0 × 10^−7^ M. In conclusion, the biosensor provided a simple and rapid method for the determination of Hx in fish meat.

Devi et al. developed an amperometric XOD-based Xa sensor, where a Pt electrode was modified with a zinc oxide nanoparticle (ZnO-NP)/polypyrrole (PPy) composite film. The biosensor exhibited a linear measuring range from 0.8 to 40 μM for Xa with an LOD of 0.8 μM. The sensor was successfully used in Labeo fish samples [62]. In another experiment, multiwalled carbon nanotubes (MWCNTs) and a polyaniline composite film were used for the modification of a Pt electrode. With this setup, a lower LOD (0.6 μM for Xa) could be obtained and the linear response range was 0.6–58 μM [63]. The previously presented electrode was further developed and supplemented with ZnO-NP/chitosan to form a nanocomposite film, modifying the Pt electrode [64]. The sensor showed an optimum response at a potential of 0.5 V within 4 s and linear range of 0.1–100 μM with an LOD of 0.1 μM for Xa. They also reported a Au-colloid–PPy nanocomposite film-modified Pt electrode for Xa determination, with a linear range from 0.4 to 100 μM for Xa and an LOD of 0.4 μM [65]. It was used for Xa determination in fish, chicken, pork, and beef meat samples. The sensor could be used for 200 measurements over 100 days with a 40% loss of its initial activity.

Another amperometric biosensor was constructed to detect Xa in fish, chicken, pork, and beef meat. The biosensor was based on the covalent immobilisation of XOD onto citrate-capped AgNPs deposited onto a Au electrode through cysteine SAM [31]. The biosensor exhibited a linear working range for Xa from 2 to 16 μM, with an LOD of 0.15 μM.

Dervisevic et al. developed a novel nanocomposite host matrix for XOD immobilisation in their amperometric biosensor to detect fish freshness [66]. They used a mixture of two redox copolymers (vinylferrocene (VFc) and glycidyl methacrylate (GMA)) due to their excellent electron transfer properties with MWCNTs for nanocomposite matrix development. The determination of Xa in fish samples was performed at a potential of 0.35 V by amperometry. The biosensor exhibited a good analytical performance, with an LOD of 0.12 μM and the linear ranges of detection (dividing the calibration curve into three parts) were 2–28 μM, 28–46, and 46–86 μM.

They also constructed another nanocomposite film by embedding reduced–expanded graphene oxide sheets decorated with iron oxide (Fe_3_O_4_) nanoparticles into a poly(glycidyl methacrylate-co-vinylferrocene) (P(GMA-*co*-VFc)) phase, on which XOD was immobilised for Xa determination in fish. In this case, a linear range of 2–36 μM could be obtained with an LOD of 0.17 μM [67]. To enhance sensitivity, a pencil graphite electrode (PGE) modified with electrochemically polymerised conducting polymer film was used for amperometric detection of Xa. The linear measuring range obtained was 0.3–25 μM, with an LOD of 0.074 μM [68].

Lately, a GCE covered with chitosan-PPy-AuNPs was used to enhance electron transfer in their electrochemical Xa biosensor. The sensor exhibited a linear range of 1–200 μM and an LOD of 0.25 μM when tested in fish, beef, and chicken samples [69].

Pierini et al. proposed a simple, rapid, and non-expensive square wave voltammetry-based sensor to determine Hx, Xa, and Ua in untreated fish samples using an edge plane EPPGE [27]. The sensor exhibited a linear range from 0.1 to 50 μM for Hx and Xa and from 0.1 to 25.0 μM for Ua. The LODs were 0.08, 0.06, and 0.03 μM for Hx, Xa, and Ua, respectively.

A differential pulse voltammetry-based enzymatic (XOD) biosensor for Xa determination was described applying GCE modified with MWCNTs and poly(L-aspartic acid) (poly(l-Asp)) film [70]. The sensor exhibited a linear working range of 0.001–0.004 μM and 0.005–50.0 μM, respectively, with a low LOD of 3.5 × 10^−4^ μM. It was successfully applied for Xa determination in fish meat.

Narang et al. also used MWCNTs with TiO_2_ nanoparticles to form a nanocomposite platform where XOD was immobilised to form a highly sensitive biosensor for the determination of fish freshness [71]. The impedance-based Xa sensor was stable for 60 days and showed excellent performance, with a linear range of 0.5 to 500 μM and an LOD of 0.5 μM for Xa.

In the work of Khan et al. for Xa determination, a GCE was modified with AuNPs of an incorporated poly(3,4-ethylenedioxythiophene)/polystyrene sulfonate (PEDOT:PSS) polymer [72]. The calibration curve was linear from 5.0 × 10^−8^ to 1.0 × 10^−5^ M, with an LOD as low as 3.0 × 10^−8^ M. The sensor was successfully applied in commercial fish and meat samples and even in the presence of Hx and Ua as interferents.

Wang et al. reported a DPV-based enzymatic biosensor for Xa and Hx detection. They applied a biocompatible Cu-based metal organic framework (Cu-MOF) nanofiber film on which XOD was immobilised [73]. The sensor showed high sensitivity with a wide linear range (0.01 to 10 μM) for Xa and Hx and was applied successfully for the detection of Hx and Xa in chilled squid and large yellow croaker fish.

A novel amperometric Xa biosensor using a layer-by-layer electrode architecture on a GCE based on hybrid nanomaterials (poly(dopamine)-modified magnetic nanoparticles coated with four-generation ethylenediamine core polyamidoamine G-4 dendrimers and further decorated with platinum nanoparticles) was reported [30]. The biosensor allowed for the amperometric detection of Xa in the 50 nM–12 μM range, with an LOD of 13 nM. Table 2 summarizes the different electrochemical biosensors for Xa determination in fish and meat samples.

### 3.3. Enzyme-Based Biosensors for Volatile Nitrogen Compounds (Ammonia, Trimethylamine, and Dimethylamine)

Trimethylamine *N*-oxide (TMAO) is found in most marine fish, but its presence is negligible in freshwater fish varieties. In post-mortem, through biochemical and microbial reactions, TMAO reduces TMA, which indicates spoilage of fish meat [74]. For estimating fish freshness, an amperometric and impedimetric biosensor was developed by Bourigua et al. for detecting TMA [75]. In the experiment, a flavin-containing monooxygenase 3 (FMO-3) enzyme was immobilised covalently using conductive Ppy substituted with ferrocenyl groups. With the biosensor, the TMA content of different fish samples was determined by measuring the oxidation currents of ferrocenyl groups and was compared to the measurement of impedance related to the electrical properties of the layers. The biosensor showed high selectivity and sensitivity to TMA in real samples with an LOD of 0.4 μg/mL for TMA and a dynamic range from 0.4 to 80 μg/mL was obtained by impedance.

Mitsubayashi et al. developed an electrochemical biosensor for TMA determination using the same FMO-3 enzyme as the recognition element [76]. For the construction, the FMO-3 enzyme was immobilised on a dialysis membrane, placed on a Clark-type oxygen electrode, and covered with a supporting Nylon mesh net, and the sensor was applied in a flow injection analysis (FIA) system. For the measurement, a potential of −600 mV was applied. A linear measuring range of 1–50 mmol/L for TMA could be achieved with the established sensor. The sensor was applied for evaluating fish freshness using horse mackerel samples. Saito et al. used the same sensor setup but not in an FIA system [77]. In their system, in order to amplify the biosensor output, a substrate regeneration cycle obtained with L-ascorbic acid by coupling the monooxygenase to the reducing reagent system was used for triethylamine measurement. Oxygen consumption induced by the FMO-3 enzymatic reaction was measured at −700 mV to detect triethylamine. The dynamic measuring range of the sensor was between 0.5 and 4 mmol/L.

In the work of Mitrova et al., an enzyme-based electrochemical biosensor for the determination of TMAO was described [78]. For the sensor setup, a chimeric variant of TMAO reductase (TorA-FDH) was entrapped onto a GCE under a permselective membrane. The biosensor was evaluated using amperometry at a potential of −0.8 V. With this sensor, an LOD of 2.96 nM TMAO could be achieved.

### 3.4. Immunosensors Based on Enzyme Labelling

A screen-printed immunosensor based on a silver electrode coated with single-walled carbon nanotubes (SWCNTs) was developed for His determination in fish samples [79]. In the sensor, anti-His antibodies were directly immobilised on an oxygen plasma-treated SWCNT-modified working electrode. For His determination, HRP-labelled His competed with His present in the sample for the binding site of the immobilised antibody. For the electrochemical measurement, the three electrodes were immersed into a buffered solution of 3,3′,5,5′-tetramethylbenzidine (TMB) and H_2_O_2_, the reduction of which was catalysed by HRP. Subsequently, the oxidised HRP was reduced by oxidizing TMB. The electrochemical current was measured by chronoamperometry at a potential of 100 mV. The developed immunosensor showed a wide linear detection range from 0.005 to 50 ng/mL for His in samples with a very low LOD of 2.48 pg/mL and high selectivity (less than 3% response for Cad, Put, and Tra).

A sensitive and selective electrochemical immunosensor was constructed to detect His by assembling a Prussian blue chitosan–AuNP nanocomposite (PB-CS-AuNP) film on an SPCE coated with a His-ovalbumine conjugate. His was determined by applying a typical competitive immunoassay; thus, the measured current was inversely proportional to the His content. The developed electrochemical immunosensor showed a linear range from 0.01 to 100 μg/mL for His in fish samples, while good specificity, reproducibility, and stability could be achieved, indicating its potential application in His monitoring in a simple and inexpensive way [80].

### 3.5. Selective Determination of Bacteria with Sensors

There are about 30 bacterial genera commonly associated with raw meat and fish. However, among these, the most common foodborne pathogens that are responsible for foodborne illnesses and spoilage are the *Escherichia coli* O157:H7, *Salmonella enterica*, *Staphylococcus aureus*, *Listeria monocytogenes*, *Campylobacter jejuni*, *Bacillus cereus*, and *Vibrio* species. The commonly used microbiological methods for pathogen detection are based on enrichment, filtration, and incubation steps; thus, the results are available within 2 to 10 days [81]. The results could be expressed in colony-forming units (CFU/mL), which estimate the number of vial microbial cells in the sample or cells/mL where all cells (dead or living) are counted. Although other methods such as immunological or nucleic acid-based methods, including enzyme-linked immunosorbent assay (ELISA) and methods that utilize polymerase chain reaction (PCR), are reliable and really sensitive, they still require time, trained personnel, and laboratories for determination. In contrast, new methods based on biosensors offer an improvement in rapidity, sensitivity, specificity, and suitability for in situ analyses. The low permitted limits of microorganisms and the toxins and metabolites they produce emphasize the need for very sensitive analytical methods for the detection of pathogens in meat [82]. For this reason, studies on novel immunosensors such as DNA- or aptamer-based sensors have been published recently.

Fei et al. developed a sandwich-type electrochemical immunosensor for *Salmonella pullorum* and *S. gallinarum* determination [24]. In their application, an SPCE electrode modified with electrodeposited AuNPs with anti-*S. pullorum* and anti-*S. gallinarum* antibodies immobilised on its surface was applied. During the measurement, a secondary antibody labelled with HRP was added after introducing the sample to the surface. After adding H_2_O_2_ and thionine as an electron mediator, the signals were measured by CV. The linear measuring range for both species was obtained in a concentration range of 10^4^ to 10^9^ CFU/mL, with an LOD of 3.0  ×  10^3^ CFU/mL.

Che et al. used a Tyr-modified enzyme electrode coupled with an enzyme-linked immunoassay for the rapid detection of Campylobacter jejuni [83]. A competitive immunoassay format was used for Campylobacter detection. In their measurement, streptavidin-labelled magnetic beads were coated with biotin-labelled rabbit anti-C. jejuni antibodies, and for the preparation of the secondary antibody-coated beads, sheep anti-rabbit IgG was used. After immunomagnetic separation, amperometric detection (at −0.15 V) was used for C. jejuni detection. The sensor was used to detect C. jejuni in chicken carcasses washed with water and ground turkey meat samples, with an LOD of 2.1 × 10^4^ CFU/mL.

An aptamer-based electrochemical biosensor was constructed for *S. typhimurium* determination in pork meat [84]. A GCE modified with graphene oxide (GO) and AuNPs was used for the sensor setup, where a thiolated DNA aptamer sequence of *S. typhimurium* was immobilised. EIS was used for *Salmonella* quantification. The linear measuring range was 2.4–2.4 × 10^3^ CFU/mL, with an LOD of 3 CFU/mL.

Sheikhzadeh et al. demonstrated a real label-free impedimetric detection method in *S. typhimurium* [85]. The sensor consisted of a combination of Ppy-based polymer and aptamers. With the established aptasensor, *S. typhimurium* could be detected in the concentration range of 10^2^–10^8^ CFU/mL, with an LOD of 3 CFU/mL.

Simultaneous, multiple DNA-based detections of *S. enteritidis* and *Bacillus anthracis* were reported by Zhang et al. [86]. The working principle of their sensor was the following: the first target-specific DNA probe that could recognize one end of the target DNA was coated with AuNPs and nanoparticle tracers (NTs) terminating ssDNA bio-barcodes as electrochemical indicators. The second target-specific DNA probe that could recognize the other end of the target DNA was coated with magnetic nanoparticles. After adding the target DNA from the samples, it formed a sandwich structure with the probes and hybridisation could begin. After hybridisation was complete, the sandwich complexes formed were separated from the unreacted probes by applying a magnetic field. The NTs were released in 1 M nitric acid and were measured by square wave anodic stripping voltammetry (SWASV) on an SPCE chip. The electrochemical signal of released NT ions increased with increasing DNA concentration in the sample. An LOD of 0.5 ng/mL for *S. enteritidis* could be achieved and for *B. anthracis*, it was 50 pg/mL.

For *Campylobacter* determination, the first electrochemical genosensor based on thin-film Au electrodes deposited onto cycloolefin polymer (COP) substrates was described and used for the control of raw poultry meat samples [87]. The analytical method relied on a Au electrode modified with a microscale thin-film on COP substrates and modification of the surface with a DNA thiolated probe. The linear measuring range was between 1 and 25 nM and the LOD was 90 pM for the concentrations of PCR amplicons of *Campylobacter*.

A real-time potentiometric biosensor for *S. aureus* determination was reported using a network of SWCNTs functionalised with *S. aureus*-binding DNA aptamers for label-free detection [88]. With the established sensor, an LOD of 800 CFU/mL could be obtained and the dynamic measuring range was 8 × 10^2^ CFU/mL to 10^8^ CFU/mL. The sensor was tested on real samples from freshly excised pig skin.

A CV-based electrochemical immunosensor was presented for the simultaneous detection of *E. coli* and *Enterobacter sakazakii* by Dou et al. [89]. In the sensor setup, HRP-labelled antibodies of the two bacteria were immobilised on the surface of the SPCE coated with an MWCNTs/sodium alginate (SA)/carboxymethyl chitosan (CMC) composite film. The linear ranges of *E. sakazakii* and *E. coli* were from 10^4^ to 10^10^ CFU/mL, with LODs of 4.57 × 10^3^ CFU/mL and 3.27 × 10^3^ CFU/mL, respectively. Table 3 illustrates the electrochemical biosensors for various foodborne pathogens.

## 4. Milk and Dairy Products

Milk is a highly complex medium containing water, carbohydrates in the form of lactose, fats, proteins (casein), citrate, mineral salts, and vitamins. These components make milk an ideal medium for the growth of microorganisms that can spoil the product. Spoilage of milk and milk products results from microbial growth or the release of extracellular and intracellular enzymes (oxidases, polymerases, proteases, esterases, lipases). Spoilage of milk can be caused by numerous different bacteria. Among them, psychotrophs comprise the largest percentage of bacteria in milk and cause spoilage at refrigerator temperatures at or below 7 °C. Acid-forming bacteria such as lactic acid bacteria (LAB, e.g., *Streptococcus Lactis*, *Lactobacilli*, *Lactococcus)* and coliforms ferment lactose to lactic acid, causing the acidification of milk, which depresses the pH to a point (about pH 4.5) where curdling takes place. Gas-forming bacteria can produce significant amounts of different gases (CO_2_, H_2_) via lactose fermentation. Thermoduric psychotrophs, capable of surviving pasteurisation or other heat treatment procedures, can cause spoilage even when pasteurised milk is stored at low temperatures [90,91,92]. For the control of dairy products, the most important analytical tasks are the determination of lactate, BAs, and bacterial species.

### 4.1. Enzyme-Based Biosensors for Lactate Determination

Lactate is one of the most important markers for bacterial fermentation, commonly used for milk quality assessment. The lactate level of fresh cow milk is about 1–2 mol/L. Under improper storage conditions (at elevated temperature) due to bacterial contamination, the lactose content of milk is converted to lactic acid [93].

Phamonon et al. presented a novel biosensor for lactate determination using ultra-high-temperature (UHT)-processed milk carton waste [94]. For the sensor construction, lactate oxidase was immobilised on an aluminium-coated cellulose working electrode which was derived from the UHT milk carton. The amperometric detection of lactate was carried out at an applied potential of +0.3 V. With the established sensor, a linear range of 0.125–2 M and an LOD of 0.23 M for the lactate could be achieved. It was used to distinguish between normal and spoiled milk in real milk samples with satisfactory results when compared with results obtained by HPLC.

Another lactate oxidase-based amperometric biosensor for L-lactate determination in food samples was described by Zanini et al. [95]. In the biosensor, lactate oxidase was immobilised on a GCE modified with laponite/chitosan hydrogels. The low applied potential (400 mV) and permeability properties of the film allowed for the interference-free determination of L-lactate. The biosensor was successfully used for the quantification of L-lactate in wine, beer, and fermented milk. The LOD of the L-lactate in wine, beer, and fermented milk was (4.1 ± 0.3) × 10^−6^ M, (3.2 ± 0.4) × 10^−6^ M, and (9.0 ± 0.5) × 10^−6^ M, respectively.

Rahman et al. developed an amperometric lactate biosensor based on lactate dehydrogenase (LDH) immobilisation on a Au electrode modified with a conducting polymer and an MWCNT composite film [96]. Chronoamperometric experiments were carried out by applying a potential of 0.3 V. The calibration curve was linear over a range of 5 to 90 μM for lactate with an LOD of 1 μM. With the established sensor, L-lactate concentration in commercial milk and human serum samples was measured.

### 4.2. Enzyme-Based Biosensors for Biogenic Amines

BA content in milk and dairy products, e.g., cheese, is related to the milk yield, lactation period, and type of cow [97,98].

The BA content in cheeses is affected by various factors such as the milk protein content, bacterial occurrence, use of starter cultures and enzymes, condition of ripening, thermal treatment, and storage conditions [99]. The BA content of cheese is higher than that of milk and it increases with applied maturation time [100].

An amperometric biosensor for the determination of total BA content by using diamino oxidase entrapped by glutaraldehyde onto an electrosynthesised bilayer film was presented [101]. In this experiment, several electroproduced anti-interferent mono- and bi-layer films were tested in order to reduce interference. In the first case, on a Pt electrode, an overoxidised poly-pyrrole film was formed on which a poly-*ortho*-phenylenediamine (PPD) film was electrochemically deposited. In the second case, on an overoxidised poly-pyrrole film, a poly-*β*-naphthol (P*β*NAP) film was allowed to grow. In both cases, the sensors were successfully used to test the presence of BAs in spicy and mild caciocavallo cheese and anchovy samples.

A novel BA biosensor was demonstrated based on indium tin oxide nanoparticles and a Prussian blue-modified SPCE electrode, on which MAO or DAO enzymes were immobilised for Cad and His determination in cheese [102]. In the case of the MAO-functionalised biosensor, the linear working range for Cad was 3.0 × 10^−6^–1.0 × 10^−3^ M (*R*^2^ = 0.99) with an LOD of 8.9 × 10^−7^ M, while the DAO-based biosensor exhibited the highest response to His in a concentration range of 6.0 × 10^−6^–6.9 × 10^−4^ M. The performance of the BA sensor was successfully demonstrated in real cheese samples with excellent recoveries of 101 to 103% for His and 103 to 105% for Cad.

Erden et al. reported an SPCE electrode modified with GO and polyvinylferrocene (PVF) for Tra determination in cheese using MAO and DAO enzymes [103]. The presented amperometric biosensors, especially the DAO sensor, showed good analytical characteristics such as a short response time (<50 s), good sensitivity, wide linear range (9.9 × 10^−7^ to 1.2 × 10^−4^ M), and low LOD (4.1 × 10^−7^ M) towards Tra determination.

Dalkiran et al. used an SPCE electrode modified with Tyr, PLL, and an Fe_3_O_4_ nanoparticle chitosan composite for Tra determination. The enzymatic product was detected by amperometry at −0.2 V. The LOD was 7.5 × 10^−8^ M and the dynamic measuring range of Tra was 4.9 × 10^−7^ M −6.3 × 10^−5^ M. With the established amperometric biosensor, the Tra levels of different cheese samples were determined [33].

Calvo-Pérez et al. developed an SPCE for the determination of Tra via flavin adenine dinucleotide-containing plasma amine oxidase. In order to lower the working potential (+260 mV), hydroxymethyl ferrocene was used as a mediator [46]. The calibration curve was linear in the Tra concentration range from 2 to 164 μM and the LOD was 2.0  ±  0.18 μM. The biosensor was successfully applied to the determination of Tra in cheese.

An amperometric Xa biosensor based on a PVC membrane-immobilising XOD was reported [104]. The current was measured at a potential of 0.4 V. The linear measuring range could be obtained in a range from 0.025 to 0.4 × 10^−6^ M for Xa with an LOD of 2.5 × 10^−8^ M. The sensor was tested in real samples (fish, meat, cow, and buffalo’s milk). It could be used about 100 times over a period of 45 days with only a 30% loss of its initial activity. The different amperometric biosensors for biogenic amine determination in dairy products are summarised in Table 4.

### 4.3. Selective Determination of Bacteria with Sensors in Dairy Products

An SPCE electrode-based electrochemical immunosensor was developed for *S. typhimurium* LT2 determination using AuNPs as labels and magnetic particles for preconcentration [105]. In this approach, *Salmonella* containing skimmed milk samples were investigated by using anti-*Salmonella* magnetic beads (MBs-pSAb) as the capture phase, followed by labelling with AuNPs modified with polyclonal anti-*Salmonella* antibodies. Afterwards, the modified MBs were captured by applying a magnetic field below the SPCE used as a transducer. For the electrochemical detection, DPV was used by scanning from +1.25 to 0 V (step potential 10 mV, modulation amplitude 50 mV, scan rate 33.5 mV/s). With this sensor setup, an LOD of 143 cells/mL and a linear range from 10^3^ to 10^6^ cells/mL of *Salmonella* was obtained.

An electrochemical immunosensor for the detection of *Salmonella* was constructed using a GCE modified with a chitosan/AuNP composite film where the selective capture antibody was immobilised for biorecognition. After incubation, an HRP-labelled secondary antibody was added and the signal was measured by DPV (−0.4 to −0.1 V, with a pulse amplitude of 50 mV and a pulse width of 20 ms). The sensor was tested by using milk samples containing *Salmonella*, where it provided a wide linear range from 10 to 10^5^ CFU/mL with a low LOD of 5 CFU/mL [106].

A multiwalled carbon nanotube-polyallylamine modified SPE (MWCNT-PAH/SPE) electrode-based immunosensor was developed for the simultaneous determination of *E. coli* O157:H7, *Campylobacter*, and *Salmonella* in milk samples [26]. The immunosensor was fabricated by immobilizing the mixture of antibodies on the surface of the modified electrode. After incubating the sensor in the sample, antibodies conjugated with three different nanocrystal tracers (CuS, PbS, and CdS) were used as secondary antibodies to perform a sandwich immunoassay. Square wave anodic stripping voltammetry (SWASV) was employed to measure released metal ions from bound antibody nanocrystal conjugates. With the established sensor, the dynamic measuring range was 1 × 10^3^–5 × 10^5^ cells/mL for the three selected bacteria with an LOD of 400 cells/mL for *Salmonella*, 400 cells/mL for *Campylobacter*, and 800 cells/mL for *E. coli*.

Bhardwaj et al. developed a label-free, low-cost, paper-based electrochemical biosensor for *S. aureus* determination. In the sensor, anti-*S. aureus* antibodies coupled with SWCNT were immobilised on a carbon working electrode [107]. DPV was used to detect *S. aureus* by analysing the change in current following antigen–antibody complex formation. The linear working range of the sensor was between 10 and 10^7^ CFU/mL, with an LOD of 13 CFU/mL in spiked milk samples.

In the work of Wu et al., a graphene-wrapped copper (II)-assisted cysteine hierarchical structure was used for the modification of a Au electrode, on which monoclonal antibodies against *S. aureus* were immobilised [108]. EIS was applied to detect *S. aureus.* With the established sensor, a linear working range of 10–10^8^ CFU/mL could be obtained with an LOD of 4.4 CFU/mL.

A disposable amperometric immunosensor was developed using Au/SPEs modified with *Staphylococcal* ProtA-functionalised MBs for the detection of *S. aureus* [109]. Competitive immunoassays involving the ProtA antigen labelled with HRP was performed. Modified MBs were captured on the surface of tetrathiafulvalene-modified Au/SPE (TTF-Au/SPEs) by a magnetic field. Amperometric measurements were carried out at room temperature at an applied potential of −0.15 V. *S. aureus* determination in milk samples was carried out with the sensor where a unique LOD of 1 CFU/mL could be achieved without any sample pre-treatment.

Majumdar et al. described an amperometric immunosensor for the detection of *S. aureus* in milk and cheese samples [110]. Here, a Pt electrode modified with a polyethyleneimine (PEI) layer was used for antibody immobilisation in the construction of the immunosensor. In real samples, for *S. aureus*, the linear measuring range was 10^1^–10^8^ CFU/mL, with an LOD of 10 CFU/mL.

Izadi et al. introduced an electrochemical DNA-based biosensor for *B. cereus* determination in milk and infant formulas by immobilisation of the single-stranded DNA (ssDNA) of the *nheA* gene on the surface of a AuNP-modified pencil graphite electrode [111]. After hybridisation, *B. cereus* was detected by using EIS. The biosensor detected *B. cereus* as low as 1 CFU/mL. Table 5 illustrates the electrochemical biosensors for the detection of spoilage-causing bacteria in dairy products.

## 5. Monitoring of Spoilage in Alcoholic and Non-Alcoholic Beverages

In addition to meat, fish, and dairy products, BAs are found also in many alcoholic and non-alcoholic beverages (wine, beer, fruit juice), so the concentration of BAs, especially His and Tra, can be used to evaluate the freshness of some beverages due to their microbiological origin [49].

### 5.1. Enzyme-Based Sensors for Alcoholic and Non-Alcoholic Beverages

For the determination of His in beverages, different types of biosensors were investigated, but all of them used MIPs as recognition elements for the biosensors. A new potentiometric biosensor for His quantification was developed in which solid-phase MIP particles were incorporated into a membrane of an anion-selective electrode. The ISE sensor could selectively determine the amount of His in the presence of other BAs in wine. The LOD reached 1.12 × 10^−6^ mol/L and the measuring range was between 10^−6^ and 10^−2^ mol/L with a response time of 20 s [22]. It was concluded that the use of nanoparticles with high specificity and affinity in the electrode matrix enabled the label-free detection of His in real samples [37].

A MIP-based electrochemical sensor was also developed to determine His, where AuNPs were electrodeposited onto the surface of a GCE and then, the electrode was modified with an L-cysteine self-assembled film through Au–S covalent binding. This amine layer facilitated the directed adsorption of the MIP layer onto the AuNP-based electrode, which was prepared by electropolymerisation using His as a template molecule and p-aminobenzenesulfonic acid (p-ABSA) as a functional monomer. The measurement was carried out using DPV as a detection method. The linear measuring range of the His biosensor was between 1.0–40.0 μM and 40.0–107.0 μM, with an LOD of 0.6 μM (LOD = 3S/N). The sensor was successfully applied to detect His in different alcoholic beverages (beer and wine) with recoveries ranging from 89.6 to 100.2% (RSD = 0.4 to 5.4%) and 87.4 to 103.9% (RSD = were 0.1–0.8%), respectively [28].

Since *Leuconostoc mesenteroides* bacteria have a high potential for the production of His and Tra in wine, a voltammetric biosensor assay using MIPs was developed for the detection of His. MIP beads were prepared by a precipitation polymerisation process and attached to the electrode surface by sol–gel immobilisation. The biosensor was used by adsorptive SV in differential modes, with a chosen enrichment time of 5 min. Under these conditions, an LOD of 0.19 μg/mL (1.0 μM) was achieved, with a linear response between 0.5 and 6.0 μg/mL (2.71–32.4 μM). Principal component analysis proved that the MIP receptor was selective for His [25].

Tra is a BA found mainly in fermented foods such as wine. Spoilage throughout the wine-making process can increase Tra concentrations, resulting in a harmful product for consumers [112]. To determine the Tra content of wines, a new functionalised polymer film was prepared by polymerisation of 4-mercaptophenylacetic acid (MPAA) and using CV. The tyrosinase enzyme was immobilised on carboxylic groups and DPV was used as the electrochemical measuring technique, resulting in an LOD and a limit of quantification (LOQ) of 3.16 μmol/L and 10.52 μmol/L, respectively [113].

Lactic acid bacteria are responsible for secondary, so-called malolactic fermentation following alcoholic fermentation and for the production of lactic acid. This process is useful and necessary during winemaking; however, by overgrowth, it significantly affects the quality of fruit juices. Loaiza et al. developed a new amperometric lactate biosensor by covalently immobilising lactate oxidase with polyethyleneimine (PEI) and glutaraldehyde as crosslinkers onto SPCEs modified with PtNPs supported on graphitised carbon nanofibers (GCNFs) [114]. The high catalytic activity of PtNPs against H_2_O_2_ and the property of ensuring the electron transfer of GCNFs enabled the development of an electrochemical lactate biosensor with increased sensitivity. The linear measurement range of the novel biosensor was 0.01–2.0 mM of lactate (with a slope of 41,302 ± 546 μA/M cm^2^), while the LOD was 6.9 μM. The analytical results of wines and ciders with only an adequate sample dilution indicate that the methods have no systematic errors. The simplicity, cheap production possibility, long-term stability, and excellent analytical characteristics of the developed biosensor make it suitable for use as an alternative to the traditional lactate determination methods used in the wine industry.

Anik et al. demonstrated a Xa biosensor prepared by electrochemical immobilisation of the XOD enzyme onto CPE via the entrapment of bismuth ions (Bi^3+^) [115]. After optimisation of the sensor, two linear ranges between 0.02 and 0.06 and 1–7.5 μM could be obtained for Xa with an LOD of 1.30  ×  10^−8^ M. With the established sensor, the Xa content of wine, energy drinks, and peach and sour cherry juice was determined.

### 5.2. Selective Determination of Bacteria with Sensors in Alcoholic and Non-Alcoholic Beverages

Biosensors were not only developed to determine by-products produced by microorganisms, but in many cases, the microorganism itself can also be detected with appropriate selectivity and sensitivity as listed in Table 6. A label-free potentiometric immunosensor was developed for the detection of *S. typhimurium* in commercial apple juice samples. The selective antibody was immobilised on a polymeric inclusion membrane containing AuNPs, which was used as a pipette tip-type GCE measurement electrode. Antibody–antigen binding was detected via a marker ion by sensing the ion flux-blocking effect without any extra steps caused by the antigen–antibody reaction. The LOD was 6 cells/mL [23].

Another selective and sensitive immunosensor for the detection of *S. typhimurium* was developed using a specific surface antigen, the porin-type outer membrane protein D (OmpD) as a biomarker. The produced anti-OmpD antibodies were attached to SPCEs modified with G–GO. The changes in the response signals obtained by impedimetric spectroscopic measurement indicated the concentration of the *S. typhimurium* serovariant. The developed immunosensor was successfully used to test the contamination of spiked water and apple juice samples. The LODs of the spiked water and juice samples was 10^1^ CFU/mL, while very low cross-reactivity with other strains proved the selectivity [38].

A new disposable amperometric immunosensor was developed for the detection of a yeast species, *Brettanomyces bruxellensis*, responsible for the spoilage of red wine. To create the sensor, SPCEs were first coated with a grapheme oxide hybrid nanomaterial reduced in AuNPs, which was then modified with 3-mercaptopropionic acid to enable the immobilisation of specific antibodies against *B. bruxellensis* through a carbodiimide coupling reaction. The functionalised electrode was used for the amperometric detection of *B. bruxellensis* in buffer solutions and red wine samples in the measuring ranges of 10–10^6^ CFU/mL and 10^2^–10^6^ CFU/mL, respectively, and with low LODs of 8 CFU/mL and 56 CFU/mL, respectively. The new disposable immunosensor showed high reproducibility, selectivity, and storage stability [116].

The early screening of foods, especially fruit-based drinks contaminated with Shiga toxin-producing *E. coli* (STEC) strains, is an important task in order to avoid serious health problems. An amperometric aptamer-based nanozyme sensor was developed for the detection of STEC in fruit juice, in which selected oligonucleotides were fixed on AuNPs featuring enzyme-like characteristics (nanozymes), and whether 3,5,3′,5′-tetramethylbenzidine (TMB) is o*x*idised was investigated. In the contaminated sample, the aptamers left the GNP surface and the free nanoparticles catalysed the oxidation of TMB. During the amperometric test, the oxidation of TMB was blocked by H_2_SO_4_ while the oxidised TMB was converted into an electrochemically active derivative. The LOD of the aptamer-nanozyme-based amperometric sensor was 10 CFU [117].

## 6. Conclusions

During biosensor measurements, the operation and efficiency of the sensor depends on two main components: the biological recognition part and the detector unit. The two components work together to determine the selectivity and sensitivity of the sensor, with high sensitivity, low LOD, specificity, reproducibility, and robustness being some of the properties expected from biosensors. A wide range of biorecognition elements can be used in biosensors such as enzymes, aptamers, antibodies, nucleic acids, cells, tissues, and MIPs. The selection of the biologically active substance depends on the analyte to be determined and the related detector, which can include a wide range of sensing units, with the most common being optical and electrochemical sensors.

The development of electrochemical biosensors is diverse, as it includes traditional techniques such as potentiometric and various voltammetric measurements, amperometric, and conductometric measuring cells, as well as EIS- and various field-effect transistor-based applications. The possibilities of application are further expanded by electrode modification—as used in the third generation of biosensors—promising new developments such as C nanotubes, C nanowires, AuNPs, TiO_2-_NPs, and the analytical application of magnetic nanoparticles. By taking advantage of these developments, a number of biosensors has been successfully developed and applied for food safety.

In this review, we surveyed the past 20 years of development of biosensors which can be successfully applied to various branches of the food industry, with special attention paid to the deterioration of meat, fish, and their products, and to checking their freshness throughout the food chain, from farm to fork. Approximately 20% of the meat produced worldwide is wasted due to spoilage, which is a combination of complex chemical, enzymatic, microbial, and environmental processes. Numerous compounds are produced during these processes which can be used as indicators of quality deterioration, such as BAs, and particularly His, Put, and Tra. A wide range of biosensors has also been developed to detect Xa and volatile compounds such as ammonia, TMA, and dimethylamine for the selective determination of bacteria.

Controlling the deterioration of milk and milk products is also important, as milk can serve as a complex medium for the growth of various microorganisms. An important indicator for determining quality can be the examination of lactate concentration, which is why numerous biosensors have been developed to determine lactate. Certain BAs also play an important role in the examination of dairy products, as well as in the selective determination of bacteria. BAs can also be found in many alcoholic and non-alcoholic beverages such as wine, beer, and fruit juice, so the freshness of some drinks can be evaluated based on the concentration of BAs, especially His and Tra, due to their microbiological origin.

Biosensors were introduced in the 1960s and have evolved greatly since then. The market for enzyme-based amperometric biosensors is moving towards achieving a zero eco-impact of the biosensor platform, and cost-effective and reliable biosensors that can be used in many fields from precision and remote medicine to smart food packaging. Although the possibilities for use are rather broad, the vast majority of biosensors on the market are used for healthcare applications, primarily in serum glucose determination for diabetes patients. The global biosensor market was estimated to be worth USD 27.5 billion in 2021 and is forecast to grow by 8% annually between 2022 and 2030. In turn, the market for biosensors will reach USD 49.6 billion by 2030 globally, primarily explained by the increasing spread of non-invasive biosensors. From a technical point of view, the market is dominated by electrochemical-based platforms. For example, in 2021, they obtained the largest revenue share of approximately 71.1%, presumably due to the simplicity of their biochemical measurement protocols and their low LODs. At the same time, excellent stability and repeatability can also be classified as advantages of electrochemical biosensors. In terms of production, advantages include robustness, the spread of new micro-production technologies, ease of handling, low cost, disposability, and independence from the turbidity of the sample [118].

However, it should be taken into account that most biosensors reported in the scientific literature are developed by academic laboratories, which rarely consider parameters such as manufacturability, cost, and biosensor performance requirements for practical applications. In reality, it is difficult for different biosensors to compete in cost with high-volume laboratory diagnostic platforms. At the same time, the time required for the result and the possibility of quick measurements on the spot increase the demand for biosensor-based technologies [119].

If food spoilage is taken into account, a significant economic result could be achieved and the amount of wasted food could be significantly reduced if the quality of food, in this case, primarily meat, fish, and dairy products, as well as spoilage processes, can be detected at an early stage with biosensors suitable for on-site testing.

## Figures and Tables

**Table 1:** Enzyme-based electrochemical biosensors for biogenic amine determination in fish and meat.

Analyte	Biorecognition Element	Electrode Material	Method	Linear Range	LOD	Sample	Reference
Cad, Put, Tra, His	Amine oxidase	MnO_2/_SPCE	Amperometry	1–50 µM 10–300 µM	0.3 µM 3.0 µM	Chicken	[47]
His	DAO	SPCE	Chrono-amperometry	1–75 mg/L	0.5 mg/L	Fish	[48]
His	DAO	SPCE	Chrono-amperometry	5–75 mg/mL	0.97 mg/L	Tuna, mackerel	[49]
His	DAO	GPH/PtNPs/Chitosan/SPCE	Amperometry	0.1–300 μM	2.54 × 10^−8^ M	Fish	[50]
Put	MAO	TTF/SPCE	Amperometry	16–101 μM	17.2 ± 4.6 μM	Anchovy	[51]
Cad Put	MAO	TTF/AuNPs/SPCE	Amperometry	19.6–107.1 mM 9.9–74.1 μM	19.9 ± 0.9 µM 9.9 μM	Octopus	[52]
His	DAO/HRP	PS/MWCNTs/Fc/SPCE	Amperometry	3 × 10^−7^–2 × 10^−5^ M	1.7 × 10^−7^ M	Fish	[53]
His, Tra, Put	MAO, TAO, DAO	Screen-printed thick-film electrode	Chrono-amperometry	200 mg/kg 200 mg/kg 100 mg/kg	10 mg/kg 10 mg/kg 5 mg/kg	Fish, meat, beer, dairy products	[54]
Spm, Spd	PAO, SPMO	PB/SPCE	Amperometry	0.003–0.3 mM 0.01–0.4 mM		Blood	[55]
Put	DAO	CeO₂NPs/GCE	Amperometry			Tiger prawn	[56]

Cad: cadaverine, Put: putrescine, Tra: tyramine, His: histamine, Spm: spermine, Spd: spermidine, SPCE: screen-printed carbon electrode, AuNPs: gold nanoparticles, PtNPs: platinum nanoparticles, GPH: graphene, TTF: tetrathiafulvalene, PS: polysulfone, GCE: glassy carbon electrode, DAO: diamine oxidase, MAO: monoamine oxidase, TAO: tyramine oxidase, PAO: polyamine oxidase, SPMO: spermine oxidase, PB: Prussian blue, MWCNTs: multiwalled carbon nanotubes, Fc: ferrocene, CeO₂NPs: ceria nanoparticles.

**Table 2:** Xanthine oxidase-based electrochemical sensors for xanthine determination.

Analyte	Biorecognition Element	Electrode Material	Method	Linear Range	LOD	Sample	Reference
Xa	XOD	Fe_3_O_4_ NP/Au	Amperometry	0.4–2.4 nM	0.4 nM	Fish	[60]
Hx	XOD	PPy/PVS/Pt	Amperometry	1.0 × 10^−7^–1.0 × 10^−3^ M	1.0 × 10^−7^ M	Fish	[61]
Xa	XOD	ZnO-NPs/PPy/Pt	Amperometry	0.8–40 μM	0.8 μM	Fish	[62]
Xa	XOD	c-MWCNTs/PANI/Pt	Amperometry	0.6–58 μM	0.6 μM	Fish	[63]
Xa	XOD	ZnO-NP/Chitosan/c-MWCNTs/PANI/Pt	Amperometry	0.1–100 μM	0.1 μM	Fish	[64]
Xa	XOD	Au-colloids/PPy/Pt	Amperometry	0.4–100 μM	0.4 μM	Fish, chicken, pork, beef	[65]
Xa	XOD	AgNPs/l-Cys/Au	Amperometry	2 to 16 μM	0.15 μM	Fish, chicken, pork, beef	[31]
Xa	XOD	Poly(GMA-co-VFc)/MWCNTs/PGE	Amperometry	2–28 μM, 28–46 μM, 46–86 μM	0.12 μM	Fish	[66]
Xa	XOD	Poly(GMA-co-VFc)/REGO-Fe_3_O_4_/PGE	Amperometry	2–36 μM	0.17 μM	Fish	[67]
Xa	XOD	DTP-alkyl-NH_2_/PGE	Amperometry	0.3–25 μM	0.074 μM	Chicken	[68]
Xa	XOD	Chitosan/Ppy/Au-NPs/GCE	Amperometry	1–200 μM	0.25 μM	Fish, beef, chicken	[69]
Xa	XOD	Poly(l-Asp)/MWCNTs/GCE	DPV	0.001–0.004 μM, 0.005–50.0 μM	3.5 × 10^−4^ μM	Fish	[70]
Xa	XOD	TiO_2/_MWCNTs/Au	Amperometry	0.5–500 μM	0.5 μM	Fish	[71]
Xa	XOD	GCE/PEDOT:PSS-AuNPs	DPV	5.0 × 10^−8^–1.0 × 10^−5^ M	3.0 × 10^−8^ M	Fish, meat	[72]
Hx Xa	XOD	GCE/Cu-MOF	DPV	0.01–10 μM	0.0023 μM, 0.0064 μM	Squid, fish	[73]

Xa: xanthine, Hx: hypoxanthine, MWCNTs: multiwalled carbon nanotubes, NP: nanoparticle, Ppy: polypyrrole, PVS: polyvinylsulphonate, c-MWCNTs: carboxylated multiwalled carbon nanotubes, PANI: polyaniline, Fe_3_O_4_ NP: iron oxide nanoparticles, AgNPs: silver nanoparticles, PGE: pencil graphite electrode, GCE: glassy carbon electrode, AuNPs: gold nanoparticles, l-Cys: L-cysteine, GMA: glycidyl methacrylate, co: copolymer, VFc: vinylferrocene, REGO: reduced–expanded graphene oxide, DTP-alkyl-NH2: 10-[4H-dithieno(3,2-b:2′,3′-d)pyrrole-4-yl]decane-1-amine, poly(l-Asp): poly(L-aspartic acid), PEDOT:PSS: poly(3,4-ethylenedioxythiophene) polystyrene sulfonate, Cu-MOF: copper-based metal organic framework, DPV: differential pulse voltammetry, XOD: xanthine oxidase.

**Table 3:** Electrochemical sensors for different foodborne pathogens.

Analyte	Biorecognition Element	Electrode Material	Method	Linear Range	LOD	Sample	Reference
*S. pullorum*, *S. gallinarum*	Anti-*S. pullorum*, *S. gallinarum*	SPCE/AuNPs	CV	10^4^–10^9^ CFU /mL	3.0 × 10^3^ CFU/mL	Egg, chicken	[24]
*C. jejuni*	Anti-*C. jejuni*/MBs	TYR/CPE	Amperometry	10^2^–10^7^ CFU/ml	2.1 × 10^4^ CFU/mL	Chicken carcass washed with water	[83]
*S. typhimurium*	*Salmonella* aptamer ssDNA	GCE/GO/AuNPs	EIS	2.4–2.4 × 10^3^ CFU/mL	3 CFU/mL	Pork	[84]
*S. typhimurium*	Amino-modified aptamer	AuDE/Ppy	EIS	10^2–^10^8^ CFU/mL	3 CFU/mL	Spiked apple juice	[85]
*S. enteritidis*, *B. anthracis*	AuNPs/1pDNA/bDNA-NT MNP-2pDNA	SPCE electrode	SWASV	–	0.5 ng/mL	–	[86]

SPCE: screen-printed carbon electrode, AuNPs: gold nanoparticles, MBs: magnetic beads, CPE: carbon paste electrode, AuDE: gold disk electrode, Ppy: polypyrrole, TYR: tyrosinase, GCE: glassy carbon electrode, GO: graphene oxide, CV: cyclic voltammetry, EIS: electrochemical impedance spectroscopy, 1pDNA: first target-specific DNA probe, 2pDNA: second target-specific DNA probe, bDNA-NT: NT-terminated bio-barcode ssDNA, SWASV: square wave anodic stripping voltammetry.

**Table 4:** Enzyme-based amperometric biosensors for biogenic amine determination in dairy products.

Analyte	Biorecognition Element	Electrode Material	Method	Linear Range	LOD	Sample	Reference
Lactate	LOx	Aluminium-coated cellulose electrode	Amperometry	0.125–2 M	0.23 M	Milk	[94]
L-Lactate	LOx	GCE/laponite/chitosan	Amperometry	4.1 × 10^−6^ M, 3.2 × 10^−6^ M, 9.0 × 10^−6^ M,	3.8 × 10^−6^ M	Wine, beer fermented milk	[95]
L-Lactate	LDH/NAD^+^	pTTCA/MWCNTs/Au	Amperometry	5 to 90 μM	1 μM	Milk, human serum	[96]
BA	DAO	Pt/PPY_ox_-P*β*NAP	Amperometry	6–100 μM	6 μM	Cheese, anchovy	[101]
Cad His	DAO	ITONP/PB/SPCE	Amperometry	3.0 × 10^−6^–1.0 × 10^−3^ M, 6.0 × 10^−6^–6.9 × 10^−4^ M	8.9 × 10^−7^ M	Cheese	[102]
Tra	DAO	PVF/GO/SPCE	Amperometry	9.9 × 10^−7^–1.2 × 10^−4^ M	4.1 × 10^−7^ M	Cheese	[103]
Tra	TYR	Fe_3_O_4_−Chitosan/PLL/SPCE	Amperometry	4.9 × 10^−7^–6.3 × 10^−5^ M	7.5 × 10^−8^ M	Cheese	[33]
Tra	PAO	SPCE	Amperometry	2–164 μM	2.0 μM	Cheese	[46]
Xa	XOD	Pt electrode	Amperometry	0.025 M– 0.4 × 10^−6^ M	2.5 × 10^−8^ M	Fish, milk	[104]

BA: biogenic amine, Cad: cadaverine, His: histamine, Tra: tyramine, Xa: xanthine, SPCE: screen-printed carbon electrode, GCE: glassy carbon electrode, MWCNTs: multiwalled carbon nanotubes, LOx: lactate oxidase, LDH: lactate dehydrogenase, pTTCA: poly-5,2′-5′,2″-terthiophene-3′-carboxylic acid, NAD^+^: oxidised form of nicotinamide adenine dinucleotide, DAO: diamine oxidase, PPY_ox_-P*β*NAP: overoxidised poly-pyrrole and poly-*β*-naphthol film, ITONP: indium tin oxide nanoparticles, PB: Prussian blue, GO: graphene oxide, PVF: polyvinylferrocene, PLL: poly-L-lysine, TYR: tyrosinase, PAO: plasma amine oxidase, XOD: xanthine oxidase.

**Table 5:** Summary of electrochemical biosensors for various foodborne pathogens in milk and milk products.

Analyte	Biorecognition Element	Electrode Material	Method	Linear Range	LOD	Sample	Reference
*S. typhimurium*	MBs-pSAb, sSAb-AuNPs	SPCE	DPV	10^3^ to 10^6^ cells/mL	143 cells/mL	Skimmed milk	[105]
*Salmonella*	SAb_1_, HRP-Ab_2_	GCE/chitosan/AuNPs	DPV	10 to 10^5^ CFU/mL	5 CFU/mL	Milk	[106]
*S. aureus*	Ab-SWCNTs	Paper-based CPE	DPV	10 to 10^7^ CFU/mL	13 CFU/mL	Milk	[107]
*S. aureus*	EcAb	rGO-Cu(II)/Au	EIS	10–10^8^ CFU/mL	4.4 CFU/mL	–	[108]
*S. aureus*	*Staphylococcal* ProtA-MBs, ProtA-HRP	TTF-Au/SPEs	Amperometry		1 CFU/mL	Milk	[109]
*S. aureus*	Human IgG, a-ProtA-Ab, a-rabbit IgG-AP	Pt/PEI	Amperometry	10^1^–10^8^ CFU/mL	10 CFU/mL	Milk, cheese	[110]
*B, cereus*	nhe A-ssDNA	AuNPs/PGE	EIS	0.02–10 nmol/L, 0.01–10 μmol/L	1 CFU/mL	Milk, formula	[111]
*E. coli,*	a-*E. coli* -mAb, a-*Campylobacter*-mAb, a-*Salmonella*-mAb a-*E. coli*-CdS, a-*Campylobacter*-PbS, a-*Salmonella*-CuS	α-MWCNT-PAH/SPE	SWASV	1 × 10^3^–5 × 10^5^ cells/mL	800 cells/mL,	Milk	[26]
*Campylobacter*					400 cells/mL,		
*Salmonella*					400 cells/mL		

SPCE: screen-printed carbon electrode, GCE: glassy carbon electrode, PGE: pencil graphite electrode, MWCNT: multiwalled carbon nanotube, MBs: magnetic nanobeads, DPV: differential pulse voltammetry, EIS: electrochemical impedance spectroscopy, SWASV: square wave anodic stripping voltammetry, MBs-pSAb: magnetic beads modified with primary antibodies specific to *Salmonella*, sSAb-AuNPs: gold nanoparticles modified with anti-*Salmonella* rabbit polyclonal secondary antibodies, SAb_1_: *Salmonella* antibody, HRP-Ab_2_: horseradish peroxidase-conjugated secondary antibody, Ab-SWCNT: anti-*S. aureus* antibody-single-walled carbon nanotube, rGO-Cu(II): reduced graphene oxide-cysteine-based copper (II) nanocomposite, EcAb: monoclonal antibody, TTF-Au/SPEs: tetrathiafulvalene-modified gold screen-printed electrode, ProtA-HRP: horseradish peroxidase-conjugated *Staphylococcal* ProtA, PEI: polyethyleneimine, a-ProtA-Ab: rabbit anti-protein A antibody, anti-rabbit IgG-AP: anti-rabbit immunoglobulin G alkaline phosphatase conjugate, nhe A-ssDNA: single-stranded DNA of nheA gene, PAH: polyallylamine, a-*E. coli*-CdS: Cd nanocrystal-tagged anti-*E. coli* antibody, a-*Campylobacter*-PbS: Pb nanocrystal-tagged anti-*Campylobacter* antibody, a-*Salmonella*-CuS: Cu nanocrystal-tagged anti-*Salmonella* antibody, a-*E. coli* -mAb: anti-*E. coli* monoclonal antibody, a-*Campylobacter*-mAb: anti-*Campylobacter* monoclonal antibody, a-*Salmonella*-mAb: anti-*Salmonella* monoclonal antibody.

**Table 6:** Summary of electrochemical biosensors for various foodborne pathogens in alcoholic and non-alcoholic beverages.

Analyte	Biorecognition Element	Electrode Material	Method	Linear Range	LOD	Sample	Reference
*S. typhimurium*	a-OmpD-mAb	SPCEs/G–GO	Impedimetry	–	10^1^ CFU/mL	Apple juice	[38]
*B. bruxellensis*	a-Bab ConA-HRP	Au-rGO/SPE	Amperometry	10^2^–10^6^ CFU/mL	56 CFU/mL	Red wine	[116]
*E. coli*	AuNPs-EC 12-31	SPE	Amperometry	10–10^9^ CFU/mL	10 CFU	Apple juice	[117]
*S. typhimurium*	a-SAb	AuNPs/PIM	Potentiometry	13–1.3 × 10^6^ cells/mL	6 cells/mL	Apple juice	[23]

SPCEs: screen-printed carbon electrode, SPE: screen-printed electrode, G–GO: graphene–graphene oxide, a-OmpD-mAb: anti-OmpD monoclonal antibodies, Au-rGO: nanogold-decorated reduced graphene oxide, a-Bab: polyclonal anti-Brett antibody, ConA-HRP: concanavalin A-peroxidase conjugate, EC 12-31: *E. coli*-specific aptamer P12–31, AuNPs: gold nanoparticles, PIM: polymer inclusion membrane, a-SAb: anti-Salmonella monoclonal antibodies.

## Data Availability

Not applicable.
